# Synthesis of ZnO-NPs Using a *Convolvulus arvensis* Leaf Extract and Proving Its Efficiency as an Inhibitor of Carbon Steel Corrosion

**DOI:** 10.3390/ma13040890

**Published:** 2020-02-17

**Authors:** Ghadah M. Al-Senani

**Affiliations:** Department of Chemistry, College of Science, Princess Nourah Bint Abdulrahman University, Riyadh 11671, Saudi Arabia; gmalsnany@pnu.edu.sa

**Keywords:** ZnO-NPs, corrosion, synthesis, carbon steel, *convolvulus arvensis* leaf extract

## Abstract

This paper studies the use of zinc oxide nanoparticles (ZnO-NPs) synthesized using an extract of *Convolvulus arvensis* leaf and expired ZnCl_2_, as efficient inhibitors of carbon steel corrosion in a 1 M HCl solution. The synthesized ZnO-NPs were characterized by Fourier-transform infrared (FTIR) and UV-Vis spectroscopy analysis. The corrosion inhibition of carbon steel in 1 M HCl was also investigated through potentiodynamic polarization, electrochemical impedance spectroscopy (EIS), and the determination of weight loss. The results show that the efficiency of the prevention increased when the concentration of ZnO-NPs was increased to 91%, and that the inhibition efficiency was still high (more than 89%) despite decreasing at high temperatures, acting as a mixed-type inhibitor. A sample of carbon steel with a protective layer of inhibitor on top was observed during immersion in 1 M HCl for 20 h; an increase in the charge transfer resistance (R_ct_) and stability of the inhibitor could be observed after 6 h. Adsorption isotherm models demonstrated that the inhibitor adsorption mechanism on the carbon steel surface followed Langmuir rather than Freundlich and Temkin behaviors. The thermodynamic parameters showed that the adsorption process is one of mixed, spontaneous, and exothermic adsorption. The results illustrate that the ZnO-NPs were a strong inhibitor of carbon steel corrosion in acid medium. The results of scanning electron microscopy (SEM) images showed that the ZnO-NPs formed a good protective film on the carbon steel surface.

## 1. Introduction

Carbon steel is an engineering material that is widely used in many industrial applications including manufacturing, construction, defense, transportation, and medicine. The corrosion of carbon steel is due to chemical or electrochemical reactions with the surrounding environment, and it is a spontaneous process [1]. Hydrochloric acid is used in many metallurgical industries, oil refineries, chemical and electrochemical processes, pickling metals, and the cleaning of some devices. The high concentrations of acid can affect the equipment and devices, causing severe corrosion [2,3]; this can affect the strength of materials, the environment, and the safety of society if no precautionary measures are taken to prevent or control it [4]. The corrosion of carbon steel may be present in different forms, such as uniform corrosion, galvanic corrosion, pitting corrosion, crevice corrosion, stress corrosion cracking, erosion corrosion, and microbial corrosion, among others.

One of the many traditional techniques available for preventing or controlling the corrosion of carbon steel is the enhancement of its corrosion resistance properties, which is achieved by the application of suitable inhibitors where corrosion occurs.

Chemical inhibitors that have used to prevent corrosion include organic compounds that contain active atoms of sulfur, oxygen, and nitrogen that bond with the metal surface to form a protective film that prevents or reduces the persistence of corrosion. These organic compounds are expensive, toxic, and hazardous to the environment. Recently, many researchers have investigated the use of environmentally friendly inhibitors. Most natural products are nontoxic, biodegradable, and inexpensive. Plant extracts contain many organic compounds that have the ability to inhibit corrosion [5], such as been demonstrated for *Bridelia ferruginea* extract [6], a methanolic extract of *Erigeron floribundus* (Kunth) [7], an oil extract of *Balanites aegyptiaca* seed [8], extracts of date palm waste [9], *Coriandrum sativum* leaf extracts [10], watermelon rind extracts [11], *Gmelina arborea* bark extracts [12], loquat leaf extracts [13], borage flower extracts [14], and Chinese gooseberry fruit shell [15].

Nanotechnology is one of the most active fields of research in modern materials science. The focus of recent research has been placed on designing and developing the biochemistry of nanoparticles using many plant extracts. Typically, they contain compounds that include electron-rich atoms similar to those found in chemical corrosion inhibitors, which are also capable of forming nanoparticles of minerals that provide a greater surface area for interaction on the carbon steel surface [14].

ZnO is one of the most promising nanomaterials due to its thermal and mechanical stability at room temperature in addition to good physical and chemical stability [16,17,18], its applications in the fields of environmental treatment and corrosion protection, and its low cost [4].

There are many studies on the use of zinc oxide synthesized using polymers [17,18] as corrosion inhibitors in different environments, and few studies on the synthesis of ZnO-NPs by green methods [16,19,20,21].

*Convolvulus arvensis* (field bindweed) is a plant of a wider family, which includes several species such as *Convolvulus althaeoides* and *Convolvulus scammonia*, which are annual or perennial herb vines. *Convolvulus arvensis* is one of the climbing plants that are currently cultivated for their aesthetic value, as it is highly prolific and able to cover large areas of building facades [22,23,24]. Some *Convolvulus* species are evergreen while some others lose their leaves seasonally; some are used in medical applications, while others are very poisonous [25].

In this study, zinc oxide was synthesized using expired zinc chloride with a *Convolvulus arvensis* leaf extract, and ZnO-NPs were characterized using Fourier-transform infrared (FTIR) and UV-Vis analysis. The efficacy of ZnO-NPs as an inhibitor of corrosion in carbon steel when immersed in 1 M HCl (standardized by a standard NaOH solution) was proved through weight loss and electrochemical measurements.

## 2. Experiment

### 2.1. Materials

All of the chemicals used in this study were of analytical grade. HCl ACS reagent, 37%, expired ZnCl_2_ reagent grade, 98%, and NaOH reagent grade, 97% were purchased from Sigma-Aldrich (Darmstadt, Germany).

### 2.2. Preparation of the Convolvulus Arvensis Extract

Fresh leaves from the *Convolvulus arvensis* leaf were collected, washed, and dried in the oven for two days at 50 °C and then cut into small pieces and stored in a glass vessel for eventual use.

The extraction was performed by adding 5 g of *Convolvulus arvensis* leaf to 100 mL of distilled water in a 250 mL conical flask and stirred for 30 min with heat (60 °C); then, the mixture was cooled and filtered through Whatman No.1 filter paper. The freshly prepared aqueous extract was used immediately after filtration.

### 2.3. Synthesis of ZnO-NPs

**Figure 1:**
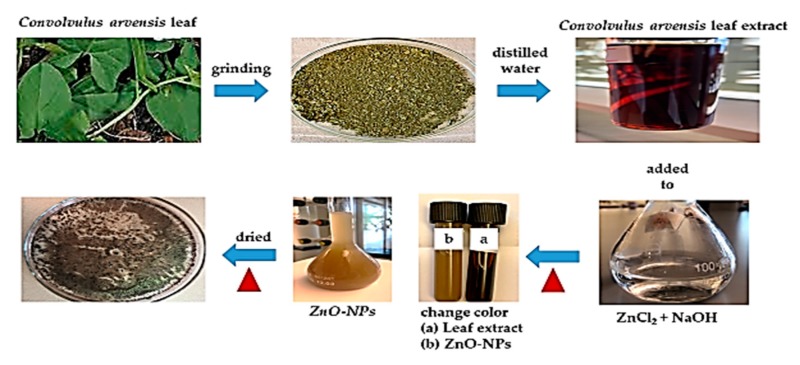
Synthesis of zinc oxide nanoparticles (ZnO-NPs).

The extract from *Convolvulus arvensis* leaf (5 mL) was added to 45 mL of expired ZnCl_2_ (0.1 M) with 0.5 M of NaOH. The solution was stirred for 30 min at 50 °C leading to a change in its color, confirming the formation of ZnO-NPs. Then, it was separated from the solution by a centrifuge, dried, and retained for later use (Figure 1).

### 2.4. Preparation of the Test Solution

One mole of a 1 M HCl solution was prepared by diluting 37% HCl with double-distilled water. Next, 300 mg of the ZnO-NPs powder was mixed with 100 mL of 1 M HCl and kept as a stock solution (pH = 2.10). All corrosion experiments, both in the presence and absence of the inhibitor at different concentrations ranging from 0.006 to 0.12 mg/mL, were carried out using this 1 M HCl solution.

### 2.5. Preparation of Carbon Steel Specimens

The sample of carbon steel used for this study was API X65 from SABIC in Saudi Arabia. The chemical composition of this carbon steel is listed in Table 1. Specimen samples were cut as cylinders with a diameter of 1 cm^2^ and inserted in a Teflon holder. Then, they were polished with 800, 1000, and 1500 grade emery papers, cleaned with acetone, washed with double-distilled water, and dried.

### 2.6. Characterization of ZnO-NPs 

FTIR spectra were recorded for the dried nanoparticles using a Fourier-transform infrared spectrophotometer (type spectrum 100 FTIR spectrometer) over a wavenumber range of 400 to 4000cm^−1^. UV-Vis analysis was carried out in the ultraviolet spectrum using a type V-770 UV-Visible/near-infrared (NIR) spectrophotometer) over a wavelength range of 200–800 nm.

### 2.7. Surface Characterization

After immersion in 1 M HCl, the morphology of the carbon steel surface was studied both in the presence and absence of 0.06 mg/mL of ZnO-NPs for 3 h at room temperature using a JSM-6380 LA model scanning electron microscope at a high resolution of 3.0 nm and an accelerating voltage of 0.5–30 kV.

### 2.8. Weight Loss Method

Carbon steel specimens were completely immersed in 50 mL of 1 M HCl solution without and with 0.006, 0.03, 0.06, 0.09, and 0.12 mg/mL of the inhibitor for 3 h at temperatures of 298 and 333 K. Then, the specimens were washed, dried, and weighed. The corrosion rates (*C*_rate_), degree of surface coverage (*θ*), and the inhibition efficiency (*E*_inh_%) were calculated from the loss in weight using the following equations:

(1) $$C_{\mathrm{rate}}=\frac{W}{At}$$

(2) $$\theta =\frac{\left(W_{0}-W_{\mathrm{inh}}\right)}{W_{0}}$$

(3) $$E_{\mathrm{inh}}\left(\%\right)=\frac{\left(W_{0}-W_{\mathrm{inh}}\right)}{W_{0}}\times 100$$

where *W* is the weight loss of carbon steel, A (cm^2^) is the area of specimens, *t* (h) is the immersion time, and *W*_0_ and *W*_inh_ are the losses in weight (mg) of carbon steel.

### 2.9. Electrochemical Measurements

A Gill AC apparatus was used to conduct potentiodynamic polarization and electrochemical impedance spectroscopy (EIS) measurements. All measurements were performed using three electrodes: the carbon steel electrode (working electrode), the graphite electrode (counter electrode), and the silver/silver chloride electrode (reference electrode). The working electrode was immersed in a test solution of 1 M HCl without and with 0.006, 0.03, 0.06, 0.09, and 0.12 mg/mL of the inhibitor at temperatures of 298 and 333 K; the open circuit potential was measured 15 min after attaining a steady state. Potentiodynamic polarization measurements were conducted at a scan rate of 0.2 mV s^−1^ and a range of ±250 mV with respect to its potential to corrode. The frequency range of EIS measurements was between 0.01 and 10,000 Hz. The inhibition efficiency (*E*_inh_ (%)) was calculated using the following equations:

(4) $$\mathrm{Potentiodynamic}\;\mathrm{polarization}:\;E_{\mathrm{inh}}\left(\%\right)=\frac{\left(i_{corr}-i_{corr\left(inh\right)}\right)}{i_{corr}}\times 100$$

(5) $$\mathrm{EIS}:\;E_{\mathrm{inh}}\left(\%\right)=\frac{\left(R_{\mathrm{ct}\left(\mathrm{inh}\right)}-R_{\mathrm{ct}}\right)}{R_{\mathrm{ct}\left(\mathrm{inh}\right)}}\times 100$$

where *i*_corr_ and *i*_corr(inh)_ are the densities of the corrosion current without and with the inhibitor, respectively, which are determined from the intercept of the cathodic and anodic Tafel slopes; and *R*_ct_ and *R*_ct(inh)_ respectively refer to the charge transfer resistance with and without the addition of the inhibitor.

All experiments were repeated three times with an error of ±0.03.

## 3. Results and Discussion

### 3.1. Characterization of ZnO-NPs

**Figure 2:**
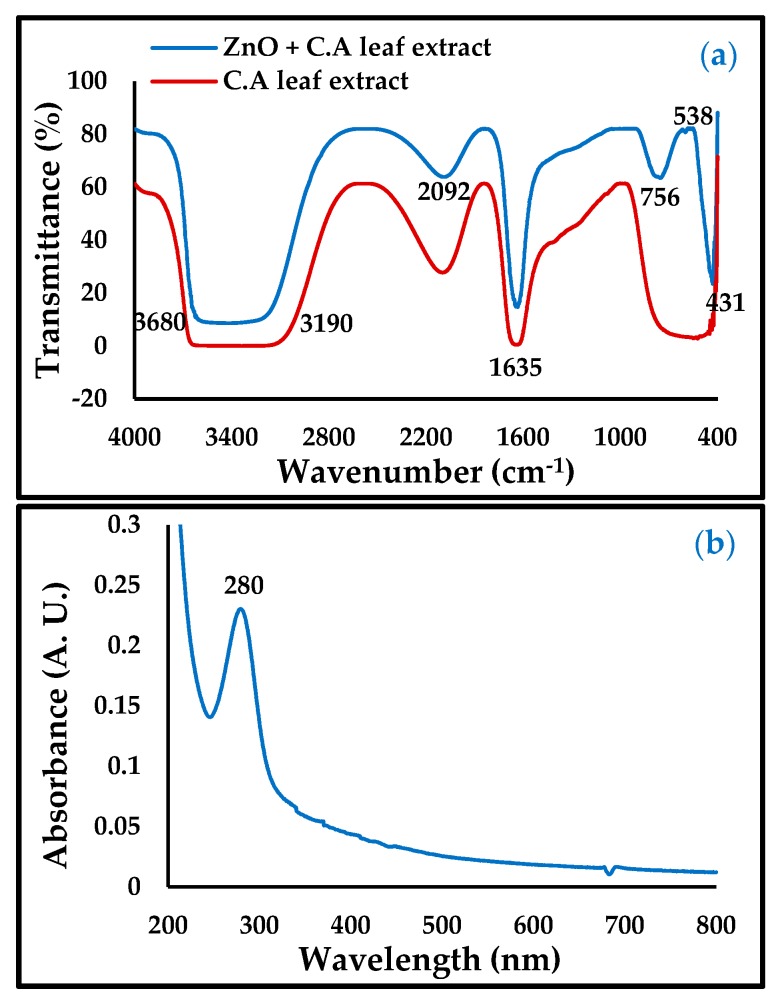
(**a**) Fourier-transform infrared (FTIR) and (**b**) UV-Vis of ZnO-NPs.

In the FTIR and UV spectra of ZnO-NPs in Figure 2, the band at 756 and 431 cm^−1^, illustrated in the UV spectrum (280 nm), is taken as evidence of the presence of ZnO-NPs. Meanwhile, the bands at 3904, 3586, 3680–3190, 2092, and 1635 cm^−1^ were attributed to the functional groups in the *Convolvulus arvensis* leaf extract [19,20]. The broad band at 3680–3190 cm^−1^ was due to the functional groups OH, NH_2_, HO-C=O, H_3_CO, and CH, and the band vibration at a height of 2092 cm^−1^ consisting of the bioactive thiamine N=C=S, while the band region in 1635 cm^−1^ is due to the presence of C=O and C=N bonds [26]. The presence of these functional groups contributes to the inhibition of carbon steel corrosion.

### 3.2. Weight Loss Method

**Figure 3:**
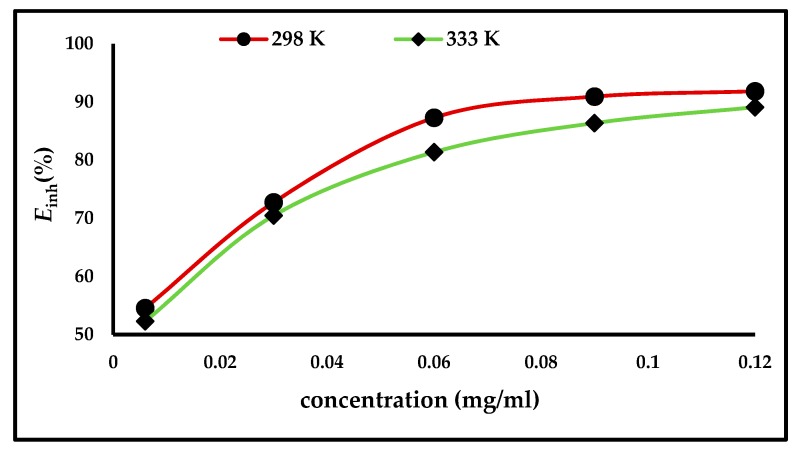
Effect of ZnO-NPs concentrations on the inhibition efficiency (E_inh_%) of carbon steel in 1 M HCl at 298 and 333 K.

Weight loss measurements were applied to evaluate the efficiency of the inhibitor, both in the presence and absence of different concentrations of ZnO-NPs. The carbon steel electrodes were immersed for up to 3 h at temperatures of either 298 and 333 K. Figure 3 illustrates that the inhibition efficiency and degree of surface coverage increases with an increase in the inhibitor concentration and becomes stable after a concentration of 0.06 mg/mL. The results are shown in Table 1, which demonstrate that the anodic and cathodic reactions are controlled by the inhibitor molecules being adsorbed on the active sites of the carbon steel surface; thus, the surface becomes saturated with the ZnO-NPs, forming a protective layer that prevents the continuation of corrosion [4,6,7].

### 3.3. Electrochemical Measurements

#### 3.3.1. Potentiodynamic Polarization Measurements

**Figure 4:**
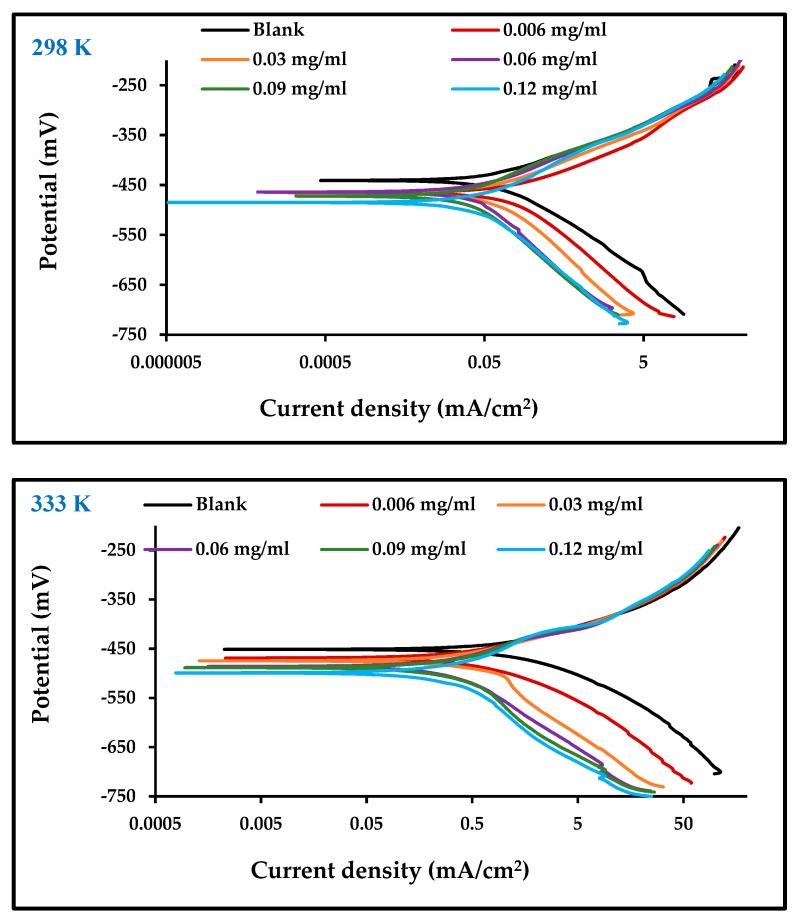
Polarization curves for carbon steel in 1 M HCl with and without different concentrations of ZnO-NPs at 298 and 333 K.

Figure 4 illustrates the potentiodynamic polarization curves for both the anodic and cathodic Tafel behavior of carbon steel corrosion in 1 M HCl in the absence and presence of different concentrations of the inhibitor at temperatures of 298 and 333 K. The Tafel slopes show that the addition of an inhibitor influences both anodic and cathodic reactions, leading to the conclusion that the effect of ZnO-NPs inhibitors is of a mixed type. A clear displacement of the Tafel anodic and cathodic curves has been observed for increases in ZnO-NPs concentration to 0.06 mg/mL and above, at which point the inhibition effect plateaus due to saturation of the carbon steel surface with inhibitor molecules [27]. It should be noted that as the concentration of the inhibitor increased, the rate of corrosion decreased. This means that the inhibitor has a high efficiency of 91% at a concentration of 0.12 mg/mL at 298 K, as recorded in Table 2. The high temperature increased *i*_corr_ [28], but the effect of the inhibitor remained the same at a temperature of 298 K. Its effect at 333 K almost became constant above ZnO-NPs concentrations of 0.06 mg/mL, showing that even at high temperatures [27], when the surface of the electrode is saturated with inhibitor molecules [29], the inhibition mechanism remains functional. The presence of ZnO-NPs, in addition to the molecules of the *Convolvulus arvensis* leaf extract on the carbon steel surface, form a protective layer that limits anodic and cathodic reactions and thus reduces the rate of corrosion [18].

#### 3.3.2. Electrochemical Impedance Measurements

**Figure 5:**
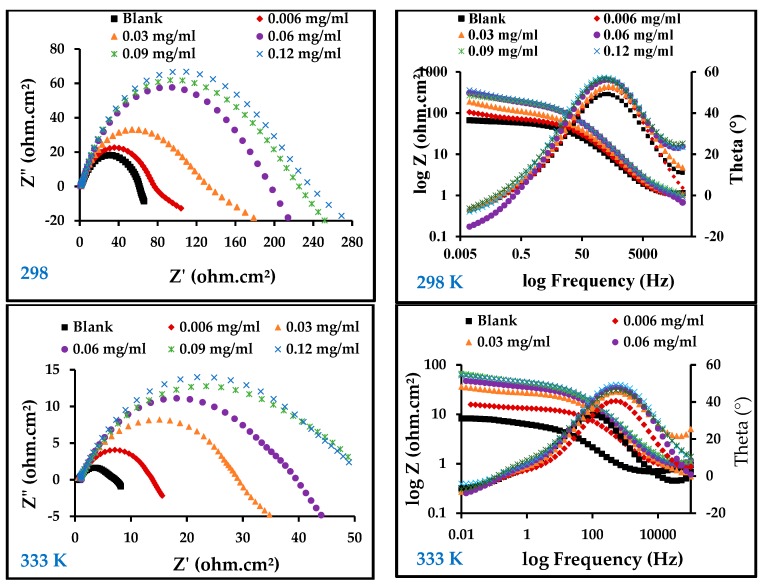
Electrochemical impedance spectroscopy (EIS) diagrams for carbon steel in 1 M HCl with and without different concentrations of ZnO-NPs at 298 and 333 K.

**Figure 6:**
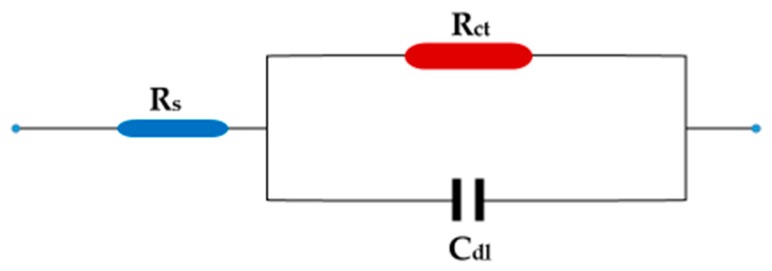
Equivalent circuit.

Figure 5 illustrates the results of an electrochemical impedance technique to determine the corrosion behavior of carbon steel in 1 M HCl in the absence and presence of ZnO-NPs at temperatures of 298 and 333 K using electrochemical impedance spectroscopy (EIS). On the Nyquist diagram of 1 M HCl compared with different concentrations of ZnO-NPs and blank solution, it can be seen that all curves follow the behavior of the blank solution. The electrochemical impedance diagram shows the presence of a semicircle of small diameter in the blank solution that increases with increasing concentrations of the inhibitor. This means that the corrosion mechanism is not altered by the inhibitor [30]. The results in Table 3 show that the double-layer capacity (*C***_dl_**) decreased with an increase in the inhibitor concentration, while the charge transfer resistance (*R*_ct_) increased with an increase in inhibitor concentration, and the corrosion rate became very low compared to the blank solution even at low concentrations. This shows that the inhibitor has a strong influence on the corrosion of carbon steel [31]. The Bode and phase angle diagrams showed increased area under the curves in the presence of the inhibitor compared to a blank solution. The corrosion resistance may be significantly increased with an increase in inhibitor concentrations [18]. The increase in *R*_ct_ and the decrease in *C*_dl_ with the increase in inhibitor concentration is due to the decrease in dielectric constant and/or increase in the thickness of the electrical double layer, which indicates that the ZnO-NPs act through an adsorption mechanism at the interface of the carbon steel/HCl solution [17]. This can be explained by substitution of the water molecules in the double layer with adsorbed ZnO-NPs, which form a protective film on the surface of the metal and thus decrease the dissolution of carbon steel [32]. Adsorption can also occur by giving or sharing non-participating electron pairs from the inhibitor molecule with the vacant orbitals on the carbon steel surface, or by bonding the inhibitor particles with the adsorption chloride ions on the carbon steel surface [4,30]. The equivalent circuit shown in Figure 6 was used for all EIS spectra; it has good compatibility with the experimental data.

The inhibition efficiency values obtained from the weight loss, potentiodynamic polarization, and electrochemical impedance measurements show good agreement with each other.

#### 3.3.3. Effect of Immersion Time

**Figure 7:**
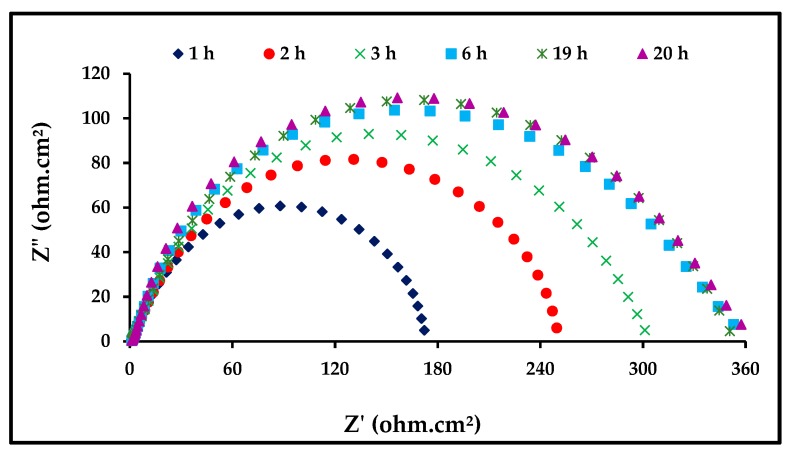
Nyquist plots for carbon steel in 1 M HCl with 6 mg/mL of ZnO-NPs at 298 K.

EIS was applied to determine the stability of ZnO-NPs with immersion time. The EIS technique studies the resistance of an electrode to corrosion without any influence on its behavior; hence, it is considered an appropriate technique for testing the immersion time. In Figure 7, we observe the response of steel to corrosion in 1 M of HCl in the presence of 0.06 mg/mL of the ZnO-NPs at different immersion durations at a temperature of 298 K. It is clear from Figure 5 that the increase in immersion time does not affect the mechanism of the corrosion process [33]. It was observed that the diameter of the semicircle in the Nyquist plots increases with an increase in immersion time. The important EIS parameters are listed in Table 4, which make it clear that *R*_ct_ increases with an increased immersion time, indicating a decreased corrosion rate. Thus, the prolonged immersion time increases the adsorption of ZnO-NPs molecules on the carbon steel surface, ensuring its stability. It was observed that the surface coverage became stable after approximately 6 hours [34,35].

### 3.4. Adsorption Isotherm Models and Thermodynamic

Various adsorption isotherm models, such as Langmuir, Freundlich, and Temkin models, were used to ascertain information about the type of reactions that occurred between the carbon steel surface and the adsorbent molecules of the inhibitor, adsorption equilibrium constant, and surface coverage.

The degree of surface coverage was determined from the data of potentiodynamic polarization. The following equations for adsorption isotherm models were applied to obtain the linear relationship between the degree of surface coverage (***θ***) and inhibitor concentration (***C_inh_***) [36,37]:

(6) $$\mathrm{Langmuir}:\;\frac{C_{inh}}{\theta }=\frac{1}{K_{ads}}+C_{inh}$$

(7) $$\mathrm{Freundlich}:\;ln\;\theta =ln\;K_{ads}+\frac{1}{n}\;ln\;C_{inh}$$

(8) $$\mathrm{Temkin}:\;\theta =\frac{-ln\;K_{ads}}{2a}-\frac{ln\;C_{inh}}{2a}$$

where K_ads_ is the adsorption equilibrium constant; a is the molecular reaction constant that attracts forces if the value is positive and repulses if it is negative; and n is a measure of adsorption intensity, where if the value of 1/n lies between 0 and 1, the inhibitor molecules are easily adsorbed on the carbon steel surface is easily, while if it is equal to 1, it is moderate, and a value greater than 1 shows that this is difficult.

**Figure 8:**
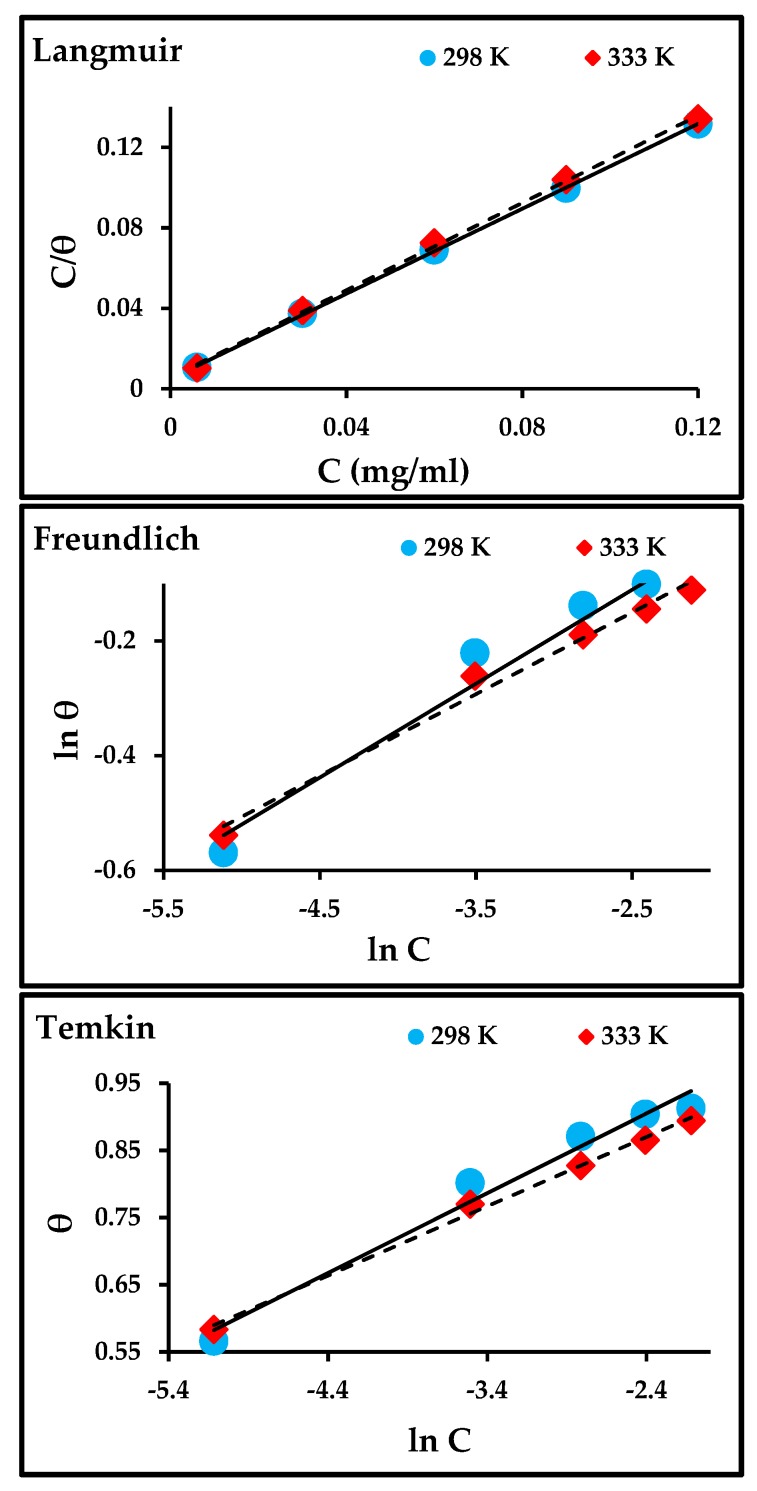
Langmuir, Freundlich, and Temkin isotherms for the adsorption of ZnO-NPs molecules on the surface of carbon steel.

The adsorption isotherm plots are presented in Figure 8, and the linear relationship and parameters obtained from those plots are listed in Table 5. The Langmuir isotherm model had the best fit when compared to Freundlich and Temkin, where the correlation coefficient (R^2^) was close to unity. The values of K_ads_ for Langmuir and Freundlich decrease with an increase in temperature, indicating that the adsorption process slows down with an increase in temperature and is unfavorable at higher temperatures. The K_ads_ for Temkin increases with an increase in temperature and suggests that the adsorption of the inhibitor on the metal surface at higher temperatures was due to physical adsorption [8,38].

Moreover, K_ads_ is also used to calculate the values of the standard Gibbs free energy (Δ*G*°_ads_) according to the equation given below [9]:

(9) $$\Delta G{{}^{\circ}}_{ads}=-\;RT\;\ln\left(55.5\;K_{ads}\right)$$

where R is the universal gas constant, T is the absolute temperature, and 55.5 is the molar heat of water adsorption. The negative Δ*G*°_ads_ values in Table 2 show that the adsorption of ZnO-NPs on carbon steel surfaces is highly spontaneous at high temperatures. The values of Δ*G*°_ads_ are between −20 and −41 kJ/mol in the Langmuir and Temkin isotherm models, which meant that both chemical and physical adsorption (mixed adsorption) occurred on the carbon steel surface. Meanwhile, it was lower than −20 kJ/mol in the Freundlich isotherm model, which meant that the adsorption of ZnO-NPs onto the surface of carbon steel was a physical adsorption process [9,39]. In general, values of Δ*G*°_ads_ less than −20 kJ/mol correspond to electrostatic reactions between the inhibitor molecules and the carbon steel surface (physisorption). Similarly, values that are lower than −40 kJ/mol involve sharing the charge or transfer from inhibitor molecules to the carbon steel surface to form a coordinate bond (chemisorption).

The adsorption enthalpy (Δ*H*°_ads_) and the adsorption entropy (Δ*S*°_ads_) for ZnO-NPs adsorbed on the carbon steel surface were calculated from the Gibbs–Helmholtz and Gibbs free energy equations [10,11]:

(10) $$\frac{\Delta G{{}^{\circ}}_{ads}\;}{T_{2}}-\frac{\Delta G{{}^{\circ}}_{ads}\;}{T_{1}}=\Delta H{}^{\circ}{}_{ads}\left(\frac{1}{T_{2}}-\frac{1}{T_{1}}\right)$$

(11) $$\Delta G{}^{\circ}{}_{ads}=\Delta H{}^{\circ}{}_{ads}-T\Delta S{}^{\circ}{}_{ads}.$$

The values of Δ*H*°_ads_ and Δ*S*°_ads_ are listed in Table 5. The negative values of Δ*S*°_ads_ are an indication that the corrosion process is controlled by an activation complex [10,11,12,32]. The negative value of enthalpies **Δ*H°_ads_*** reflect the exothermic behavior of the inhibitor on the carbon steel surface in the Langmuir and Freundlich isotherms, but this value is positive for the Temkin isotherm; moreover, the positive value of **Δ*H°_ads_*** reflects the fact that the adsorption process is endothermic [10].

### 3.5. Scanning Electron Microscopy (SEM) and Energy-Dispersive X-Ray Spectroscopy (EDS)

**Figure 9:**
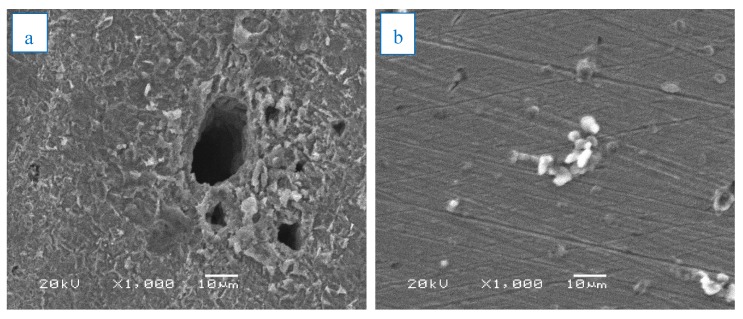

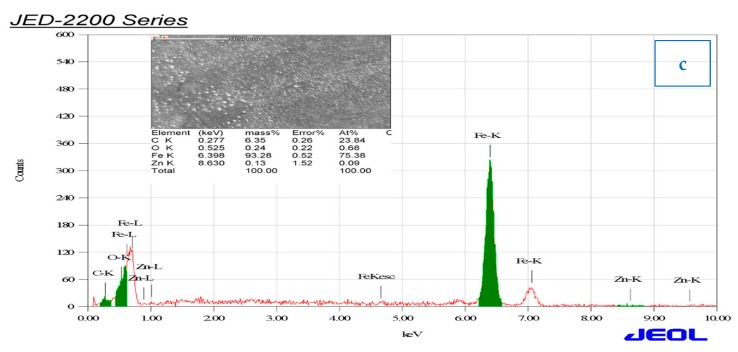
SEM and energy-dispersive spectroscopy (EDS) images of carbon steel immersed in (**a**) 1 M HCl and (**b**,**c**) also in the presence of 0.06 mg/ml of ZnO-NPs for 3 h at 298 K.

The carbon steel surface was studied by a scanning electron microscope and energy-dispersive spectroscopy after immersion for 3 h in 1 M HCl solution in the absence and presence of 0.06 mg/mL of ZnO-NPs. Figure 9a,b illustrates that the carbon steel in the blank solution was highly corroded, as cracks and pits appeared on the surface along with scratches, while in the presence of an inhibitor, corrosion was prevented and the surface was free of pits and cracks, and very few scratches were present [39,40]. Deposits of ZnO-NPs were also observed on the surface, resulting in the formation of a protective film on the carbon steel surface. Hence, ZnO-NPs are an effective inhibitor of corrosion in carbon steel exposed to HCl solution. Figure 9c shows that the presence of the signals of O, Zn, and C atoms indicate the effect of the inhibitor on the carbon steel surface, and they also prove the binding of ZnO and *Convolvulus arvensis* leaf extract with cathodic and anode active sites on the carbon steel surface [34]. The low signal of O, high signal of Fe, and more than one signal of Zn, in addition to the carbon signal, indicate a high inhibitor efficiency in reducing the corrosion rate and protecting the carbon steel surface [41].

### 3.6. Mechanism of Corrosion Inhibition

ZnO-NPs and *Convolvulus arvensis* leaf extract dissolved in HCl are transported toward the carbon steel surface, where Fe^2+^ cations and H^+^ ions are produced on the anodic and cathodic active sites, respectively:

Fe → Fe^2+^ + 2e^−^ (anodic reaction)

2H^+^ + 2e^−^ → H_2_ (cathodic reaction).

In addition, the dissolved oxygen in HCl solution exposed to the atmosphere reduction to water, as follows:

O_2_ + 4H^+^ + 4e^−^ → 2H_2_O (cathodic reaction).

The functional groups in *Convolvulus arvensis* leaf extract, which include O, C, and N atoms, interact with ZnO and Fe^2+^, forming a complex on the interface of the carbon steel/HCl solution, as follows:

Fe^2+^ + ZnO-*Convolvulus arvensis* leaf extract complex.

This complex occupies the active anodic sites, and reduces the corrosion rate [34,42].

Generally, the adsorption mechanism of the inhibitor molecules on the carbon steel surface is as follows:

- The inhibitor molecules are adsorbed onto the carbon steel surface by electrostatic interaction between the electrons adsorbed on the carbon steel surface (physical adsorption).
- The presence of heteroatoms having free electron pair enhances the chemical adsorption.
- The carbon steel surface becomes more negative for the accumulation of electrons on it.

This contributes to the transfer of electrons from the d orbitals of Fe to the non-bonding π orbitals in the inhibitor molecules, thereby enhancing the inhibitor adsorption on the surface of the carbon steel [16,43].

## 4. Conclusions

ZnO-NPs can be prepared by synthesis using expired ZnCl_2_ and *Convolvulus arvensis* leaf extract. The results obtained from the methods of weight loss, potentiodynamic polarization, and EIS measurements demonstrated that ZnO-NPs in addition to presence of N, O, C, and S atoms in functional groups of *Convolvulus arvensis* leaf extract are an effective inhibitor of carbon steel corrosion in 1 M HCl. The inhibition efficiency increases with increasing ZnO-NPs concentration and decreases at higher temperatures. It was found that the inhibition efficiency was more than 91% at 298 K and 89% at 333 K; thus, it also works as a mixed-type inhibitor. The prolonged immersion time increases the adsorption of ZnO-NPs molecules on the carbon steel surface, and the surface coverage became stable after approximately 6 h as the R_ct_ value increased from 200 to 359.3 ohms.cm^2^, after which it stabilized. The process of carbon steel corrosion inhibition followed the Langmuir isotherm (R^2^ = 0.999) more closely than Freundlich (R^2^ = 0.960) and Temkin (R^2^ = 0.976) isotherms. The calculated values for Δ*G*°_ads_, Δ*H*°_ads_, and Δ*S*°_ads_ showed that the adsorption process was spontaneous and exothermic, and that inhibitor molecules adsorbed onto the surface of the metal as a result of the chemisorption mechanism through heteroatoms having a free electron pair, and the physisorption mechanism through electrostatic interaction between the electrons adsorbed on the surface of the carbon steel. The results of the SEM and ESD studies revealed that the ZnO-NPs can act as an effective inhibitor of carbon steel corrosion in 1 M HCl solutions, where the formation of a complex of Fe^2+^ + ZnO−*Convolvulus arvensis* leaf extract on the interface of the carbon steel/HCl solution reduces the corrosion rate.

## Figures and Tables

**Table 1:** The values of C_rate_, and E_inh_% for different ZnO-NP concentrations at 298 and 333 K in 1 M HCl solution.

Temperature	298 K			333 K		
Concentration (mg/mL)	*C*_rate_ (mg/cm^2^∙h)	*θ*	*E*_inh_ (%)	*C*_rate_ (mg/cm^2^∙h)	*θ*	*E*_inh_%
Blank	1.14	-	-	5.72	-	-
0.006	0.52	0.55	54.55	2.73	0.52	52.27
0.03	0.21	0.82	81.82	1.43	0.75	75.00
0.06	0.16	0.86	86.36	1.07	0.81	81.36
0.09	0.10	0.91	90.91	0.78	0.86	86.36
0.12	0.09	0.92	91.82	0.62	0.89	89.09

**Table 2:** Potentiodynamic polarization parameters.

Concentration (mg/mL)	298 K					333 K				
	*E*_corr_ (mV)	*β*_a_ (mV/dec)	*β*_c_ (mV/dec)	*i*_corr_ (mA/cm^2^)	*E*_inh_ (%)	*E*_corr_ (mV)	*β*_a_ (mV/dec)	*β*_c_ (mV/dec)	*i*_corr_ (mA/cm^2^)	*E*_inh_ (%)
Blank	−441.01	76.90	123.28	5.15	0	−451.83	109.87	150.38	9.93	0
0.006	−464.22	70.78	133.75	2.24	56.60	−468.61	91.34	147.17	4.29	58.35
0.03	−466.08	69.55	144.68	1.02	80.17	−480.03	71.25	145.87	2.37	77.02
0.06	−464.29	67.06	145.97	0.67	87.07	−486.48	67.38	143.65	1.78	82.76
0.09	−473.69	65.83	147.10	0.50	90.39	−491.62	66.73	137.29	1.39	86.55
0.12	−484.89	68.11	136.95	0.45	91.26	−511.89	64.75	132.25	1.09	89.47

**Table 3:** EIS parameters.

Concentration (mg/mL)	298 K			333 K		
	*R*_ct_ (ohms∙cm^2^)	*C*_dl_ (mF)	*E*_inh_ (%)	*R*_ct_ (ohms∙cm^2^)	*C*_dl_ (mF)	*E*_inh_ (%)
Blank	21.55	0.56	-	5.73	7.33	-
0.006	55.48	0.47	61.16	12.58	1.00	54.45
0.03	127.00	0.43	81.26	27.86	0.85	79.43
0.06	191.30	0.36	88.73	35.61	0.63	83.91
0.09	220.10	0.36	90.21	45.34	0.54	87.36
0.12	226.70	0.30	90.49	59.48	0.52	90.37

**Table 4:** EIS parameters.

Time (h)	*R*_ct_ (ohms∙cm^2^)	*C*_dl_ (mF)
1	200.0	0.53
2	276.4	0.49
3	302.0	0.32
6	343.4	0.30
19	353.0	0.29
20	359.3	0.28

**Table 5:** Adsorption isotherm parameters.

Isotherm Models	Temperature (K)	*K*_ads_	*R^2^*	*n*	*a*	Δ*G*°_ads_ (kJ/mol)	Δ*H*°_ads_ (kJ/mol)	Δ*S*°_ads_ (J/mol K)
Langmuir	298	200	0.9998	-	-	−23.0778	−2.66	−68.51
	333	178.57	0.9989	-	-	−25.4746		
Freundlich	298	1.35	0.9604	0.164	-	−10.6899	−2.15	−28.65
	333	1.23	0.9862	0.143	-	−11.6902		
Temkin	298	22,324.87	0.9766	-	4.205	−34.7599	19.22	−181.15
	333	50,633.28	0.9955	-	−4.845	−41.1097		
